# Distinct Hypothalamic Paraventricular Nucleus Inputs to the Cingulate Cortex and Paraventricular Thalamic Nucleus Modulate Anxiety and Arousal

**DOI:** 10.3389/fphar.2022.814623

**Published:** 2022-01-28

**Authors:** Ying Liu, Bo Rao, Shuang Li, Ning Zheng, Jie Wang, Linlin Bi, Haibo Xu

**Affiliations:** ^1^ Department of Radiology, Zhongnan Hospital of Wuhan University, Wuhan, China; ^2^ State Key Laboratory of Magnetic Resonance and Atomic and Molecular Physics, Key Laboratory of Magnetic Resonance in Biological Systems, Wuhan Center for Magnetic Resonance, Wuhan Institute of Physics and Mathematics, Chinese Academy of Sciences, Wuhan, China; ^3^ Department of Pathology, School of Basic Medical Sciences, Wuhan University, Wuhan, China; ^4^ Wuhan University Center for Pathology and Molecular Diagnostics, Zhongnan Hospital of Wuhan University, Wuhan, China

**Keywords:** paraventricular hypothalamic nucleus, glutamatergic neurons, anxiety-like behaviors, wakefulness, optogenetics, chemogenetics

## Abstract

Insomnia and anxiety are two common clinical diseases that threaten people’s physical and mental health. Insomnia and anxiety may share some similar underlying neural circuit mechanisms in the brain. In this study, we combine techniques including chemo-fMRI, optogenetics, and chemogenetics to reveal that the glutamatergic neurons of the paraventricular hypothalamic nucleus (PVN) regulate both anxiety and arousal through two different downstream neural circuits. Optogenetic activation of the PVN-cingulate cortex (Cg) neural circuit triggers anxiety-like behaviors in mice without affecting the wakefulness, while optogenetic activation of the PVN-paraventricular thalamic nucleus (PVT) neural circuit promotes wakefulness in mice without affecting anxiety-like behaviors. Our research reveals that PVN is a key brain area for controlling anxiety and arousal behaviors. We also provide a neurological explanation for anxiety disorder and insomnia which may offer guidance for treatments including drugs or transcranial magnetic stimulation for the patients.

## Introduction

Insomnia and anxiety are both clinically common diseases that endanger human health. Healthy sleep is very important for people’s physical and mental health. Sleep disturbance is highly prevalent in psychopathology, and sleep impairments are found in almost every major psychiatric disorder. For example, there is an inseparable relationship between sleep disorders and anxiety disorders. Sleep problems may lead to anxiety and depression, and anxiety is often experienced along with insomnia (Fuller et al., 1997; Staner, 2003). Sleep disorder is one of the core symptoms of mental illness such as anxiety and depression. For example, sleep quality can be used to predict anxiety in veterans with posttraumatic stress disorder (PTSD) (Mantua et al., 2018), suggesting that insomnia and anxiety disorders may share similar underlying neural circuit mechanisms. Therefore, it is of great significance to explore the similarities and differences between the neural circuits that regulate these two behaviors.

The paraventricular hypothalamic nucleus (PVN) is located in the hypothalamus, adjacent to the third ventricle. Classically, neurons in PVN are defined according to the neuropeptides they secrete, including magnocellular neurosecretory cells, parvocellular neurosecretory cells, and some centrally projecting neurons (Brooks et al., 1966; Ferguson et al., 2008). These neurons project to the pituitary gland, brainstem, spinal cord, and many different brain regions and play important roles. The parvocellular neurosecretory cells mainly secrete corticotropin-releasing hormone (CRH) and thyrotropin-releasing hormone (TRH) into blood vessels and initiate the hypothalamic–pituitary–thyroid (HPT) axis and hypothalamic–pituitary–adrenal (HPA) axis (Brooks et al., 1966). The magnocellular cells mainly project to the posterior pituitary gland and secrete two peptide hormones: oxytocin and vasopressin (Moos et al., 1984; Brown et al., 2013). PVN participates in the regulation of various activities such as autonomic nervous activity (Patel, 2000), cardiovascular activity (Patel, 2000; Pyner, 2014), circadian rhythm (Klein et al., 1983; Buijs and Kalsbeek, 2001; Tousson and Meissl, 2004; Buckley and Schatzberg, 2005), energy, and feeding behavior (Waterson and Horvath, 2015; Timper and Brüning, 2017). CRF is one of the most important neuropeptides for coordinating the adaptive response of an organism to stressful situations (Daviu et al., 2020). Abundant studies have revealed that CRH is involved in stressor-induced alterations in sleep and in the regulation of stress-induced waking (Opp, 1995; Chang and Opp, 1999; Chang and Opp, 2001; Chang and Opp, 2002; Li et al., 2020; Ono et al., 2020). As the classic excitatory neurotransmitter in the central nervous system, the function of glutamate in PVN has attracted attention in recent studies. Glutamatergic neurons of the paraventricular nucleus are critical contributors to the development of neurogenic hypertension (Basting et al., 2018). Chemogenetic activation of glutamatergic neurons that express TRH promotes food intake (Suyama and Yada, 2018). The aforementioned behaviors are motivational, aroused behaviors. But only a few studies have ever systematically studied the PVN glutamatergic neurons in regulating anxiety and wakefulness.

In previous studies, we have revealed that the glutamatergic neurons in the PVN regulate wakefulness. We used fiber photometry to reveal that PVN^vglut2^ neurons were wake-active and then used chemogenetics to elucidate PVN^vglut2^ neurons were sufficient and necessary to promote wakefulness (Liu et al., 2020). A recently published study has also illuminated the function of the PVN^vglut2^ neurons in regulating wakefulness, which was consistent with our findings (Huang et al., 2021). Furthermore, through fluorescent sections of the mouse brain, we found that the downstream brain regions of the PVN included the cingulate cortex (Cg) and paraventricular thalamic nucleus (PVT), which were related to anxiety (Liang et al., 2020) and arousal regulation (Ren et al., 2018), respectively. In this study, we use a series of techniques including chemo-fMRI, chemogenetics, and optogenetics, and prove that PVN^vglut2^ neurons can regulate both anxiety and wakefulness through two different downstream neural circuits. The PVN-PVT pathway regulates arousal but not anxiety-like behavior, while the PVN-Cg pathway is critical for regulating anxiety-like behavior but not arousal. Our study may help uncover the neuronal mechanisms for anxiety disorders and insomnia.

## Materials and Methods

### Experimental Animals

All experiments were performed in accordance with the Animal Care Committee of the Wuhan University and the Wuhan University Guide for the Care and Use of Laboratory Animals. Male rats (SD) weighing about 200–250 g at 8 weeks were used in the fMRI study. Male mice (C57BL/6) weighing about 20–30 g at 8–12 weeks were used in the sleep and anxiety experiments. All experimental animals were housed (four to five per cage) in standard laboratory cages on an automatically controlled 12 h light/dark cycle (light on at 7:00, light off at 19:00), in a temperature-controlled room (24–25°C). Food and water were freely available in the environment.

### Stereotactic Technology

The rats were anesthetized by intraperitoneal injection of 1% sodium pentobarbital at a dose of 6 µl/g, and then, the head was fixed on a stereotaxic device. The PVN coordinates (AP = −1.35 mm, ML = ± 0.2 mm, DV = −8.2 mm) according to the rat brain atlas. Bilateral PVN brain areas were injected with rAAV-CaMKIIα-hM3Dq-mCherry-WPRE-pA (150 nl/side) (BrainVTA Co., Ltd., Wuhan, China, 9-42-K190108) to label the PVNvglut2 neurons. More than 90% of PVN consists of glutamatergic neurons (Ziegler et al., 2005; Xu et al., 2013). This protocol followed the method used in the study by Ren et al. (2018), in which the adeno-associated virus (AAV) expressing the genetically encoded Ca^2+^ sensor GCaMP6f was injected under the control of the CaMKIIa promoter into the PVT to label the PVTvglut2 neurons. After 4 weeks of virus expression, a 7T NMR scan was performed, and then, the chemo-fMRI experimental operations were conducted. For the mice, they were anesthetized by intraperitoneal injection of 1% sodium pentobarbital. The coordinates refer to the PVN coordinates in which the virus was injected, PVT coordinates (AP = −1.2 mm, ML = ±0.55 mm, DV = −2.95 mm, 10°angle) according to the mouse brain atlas. A different virus was injected according to the experimental design. rAVV-CaMKIIα-hM3Dq-mCherry was used for chemogenetic activation of the glutamatergic neurons of rats in the fMRI study. The rAVV-CaMKIIα-ChR2-GFP-WPRE-pA (BrainVTA Co., Ltd., Wuhan, China, 9-297-K200702) was used for optogenetic activation of PVN-Cg and PVN-PVT neural circuits in anxiety and sleep experiments. The rAAV-DIO-hM4Di-mcherry-WPRE-pA (BrainVTA Co., Ltd., Wuhan, China, k9-20201809) and rAAV-retro-Syn-Cre-WPRE-pA (BrainVTA Co., Ltd., Wuhan, China, r-202005233a) virus were used for chemogenetic inhibition of the PVN-PVT neural circuit. For the optogenetic experimental part, the optical fiber ferrule was embedded in the target brain area, above the PVN (AP = − 0.6 mm, ML = ± 0.2 mm, DV = − 4.50 mm), PVT (AP = −1.2 mm, ML = ±0.55 mm, DV = −2.65 mm, 10°angle), and Cg (AP = +0.9 mm, ML = ±0.2 mm, DV = −1.65 mm), respectively. Behavior tests were conducted after 3 weeks.

### 7T MR Data Scanning for Rat

We tried scanning the fMRI data of mice, but the SNR was relatively low may be due to hardware factors such as coils. So we used rats to conduct the 7T fMRI studies. At the beginning of the experiment, 2% isoflurane (Reward, China) and an air mixture with 20% oxygen content were used to induce anesthesia successfully, and then, dexmedetomidine at a dose of 1 ml/kg was used to induce sedation. During scanning, the concentration of isoflurane was controlled at 0.8–1.0% to maintain anesthesia. The breathing rate of rats during the experiment was monitored by pressure-sensitive abdominal breathing pads and small animal monitoring equipment to maintain it at 60 beats per minute. The body temperature of the rats during the test was maintained at 37°C using a water bath system.

A 7.0 T BioSpec machine (Bruker, Ettlingen, Germany) was used for the acquisition of MRI images. T2-weighted imaging, four EPI sequences, and FlASH sequences were collected. T2-weighted imaging parameters were as follows: repetition time (TR) = 3000 ms, echo time (TE) = 20 ms, rare factor = 4, number of averages (NA) = 4, number of repetition (NR) = 1. EPI sequence parameters: repetition time (TR) = 2000 ms, echo time (TE) = 13.5, number of averages (NA) = 1, the number of slices is 18, number of repetition (NR) = 210. Flash sequence parameters: repetition time (TR) = 500 ms, echo time (TE) = 13.5 ms, NA = 2, NR = 50, number of slices is 18, spaceless scanning, the flip angle is 30°. When the Flash sequence was collected for 24 min, intraperitoneal injection of CNO was given through the rat’s abdominal cavity to activate the PVN glutamatergic neurons (the injection dose was 2 mg/kg, and the injection time was 1 min), the total acquisition time of Flash sequence is 80 min.

### Data Processing

The Bruker2Analyze Converter was used to convert the collected data format into the NifTI (hdr/img) format. SPM12 software, DPARSF software, and MATLAB (MathWorks, Inc., CA, United States) were used for data preprocessing and data analysis. The preprocessing of the EPI sequence and Flash sequence were carried out separately. The main steps of preprocessing included slice timing, head motion correction, spatial normalization, smoothing, detrend, and filtering. The ferritin-expressing areas were segmented based on mouse brain MRI-T1 templates and atlas images. The atlas images of the mouse brain were constructed from the Paxinos and Franklin atlas figures (Nie et al., 2013). First, every individual image of the mouse brain was co-registered with a pair of brain templates and brain atlas images, using the nearest interpolation method (Nie et al., 2013).

### Optogenetics Experiments and Analysis

In the anxiety-related experiments of different neural circuits, we injected ChR2-GFP and GFP viruses into the PVN brain area, respectively. The virus was injected using the Elite 11 Nanomite Syringe Pump (Harvard Apparatus, PC2 70-4507, United States) *via* a micropipette opening ∼20 μm. The total injection time was 8 min each time; the pipette was left in place for an additional 8 min and then slowly retracted. For optical stimulation, embedded ferrules above PVN, Cg, and PVT respectively. The fiber-optic cannula was placed at least 500 μm above the target brain area and cemented onto the skull using dental cement (Lang Dental Manufacturing): the PVN (AP = − 0.6 mm, ML = ± 0.2 mm, DV = − 4.50 mm), PVT (AP = −1.2 mm, ML = ±0.55 mm, DV = −2.65 mm, 10°angle), and Cg (AP = +0.9 mm, ML = ±0.2 mm, DV = −1.65 mm). Mice were allowed 3–4 weeks to recover and express the virus before behavioral analysis. To deliver the optical stimulation, the fiber was connected to a 473-nm laser and controlled by a function generator (Thinker Tech Nanjing Bioscience Inc., China). For optogenetic activation of the PVN, PVN-PVT, and PVN-Cg, mice received 10-ms light pulses at 10 Hz for all light-on conditions. For optogenetic LTD induction (Beattie et al., 2000; Chevaleyre and Castillo, 2003; Malenka and Bear, 2004; Kreitzer and Malenka, 2007; Peineau et al., 2007), mice were photostimulated for 30 min with trains of 473 nm light (1 Hz, 4 ms), the protocol was according to our previous study (Chen and Bi, 2019). LTD is for the sleep experiment of the PVN-PVT; we gave light stimulation lasting 120s (10 Hz, 10 ms) after the mice entered stable NREM sleep in the daytime. Each mouse was tested three times, and the average time was noted. The intensity power was about 10–15 mW.

### Polysomnographic Recording and Analysis

Mice that underwent polysomnographic recordings were implanted with EEG-EMG electrodes. The implant comprised four stainless-steel screws (two serving as EEG electrodes, the other two reference and ground screw respectively), two EEG screws were inserted into the frontal region (coordinates, bregma: AP = +1.50 mm; ML = −1.50 mm) and the lateral parietal region (coordinates, bregma: AP = −3.0 mm; ML = +2.0 mm), respectively. Two EMG electrodes with wire leads were placed between the neck musculature and then the incision was closed. All electrodes were previously soldered to a micro-pin connector. The EEG-EMG apparatus was affixed to the skull with dental cement. After 3 weeks of postoperative recovery, the mice were connected to the recording leads and placed in the experimental cages for a 2-day habituation period. EEG/EMG signals were recorded under different treatment conditions (i.e., chemogenetic stimulation). For CaMKIIα-hM4Di-mCherry mice, the saline group was administrated with saline (i.p., 1 ml/kg) at 21:00 (i.e., 2 h after light-off), and CNO group were administrated with CNO (i.p., 0.3 mg/ml/kg) at 21:00 (i.e. 2 h after light-on). The sleep state was scored offline using Sirenia Sleep Pro software (Pinnacle Technology Inc., KS, United States). The recordings were first scored automatically by 4 s epochs for the wake, NREM sleep, and REM sleep, followed by a manual assessment to confirm each epoch’s accuracy. We defined wakefulness as desynchronized EEG and high levels of EMG activity. NREM sleep was defined as synchronized, high-amplitude, low-frequency (0.5–4 Hz) EEG. REM sleep was defined as having a pronounced theta rhythm (4–9 Hz) with no EMG activity. The NeuroExplorer software was used to conduct the EEG power band analysis, delta (0.5–4 Hz), theta (4–9 Hz), alpha (9–13 Hz), beta (13–30 Hz), and gamma (30–50 Hz).

### Elevated Plus-Maze Test and Open Field Experiments

Mice underwent 60 min habituation in the testing room before the elevated plus-maze test and open field experiments.

Elevated plus-maze test (EPM): As with our previous studies (Bi et al., 2015), the test was performed on an elevated, plus-sign-shaped runway that was ∼40 cm above the floor, with two opposing closed arms (10 × 50 cm) and two open arms (10 × 50 cm) and one intersection (10 × 10 cm). Before the test, mice were allowed to acclimate to the testing room for 30 min. At the time of the test, the mice were placed in the center of the maze with their heads toward open arms and the retention time of the mice in both arms during the experimental period (5 min) was recorded. The time spent in the closed and open arms was quantified autonomously using the DigBehv animal behavior analysis software. Successful open entries or enclosed entries are defined as all the limbs of the animal are in the arm or 80% of the animal’s body is in the arm. Otherwise, cases are defined as aborted entries.

Open field experiment: The center area was illuminated by halogen bulbs (200 lux, 200 cm above the field). The test mice were placed in the experiment room for 30 min in advance to adapting to the room environment, then the mice were placed at the center of the opening experiment box (50 cm × 50 cm × 40 cm), and were observed for 5 min. The retention time in the central area was then recorded. After the experiment, the mouse excrement was cleared, and the experiment box was wiped with ethanol cotton to eliminate the smell of the previous mouse. The center time, the total path, and the speed were included in the open field experiment.

### Statistical Analysis

Statistical tests were performed using GraphPad Prism software (La Jolla, CA). Figures were drawn and adjusted using Adobe Photoshop CS6. All data are presented as mean ± SEM. Comparisons of experimental groups and control groups were analyzed by two-way repeated measures ANOVA followed by Bonferroni’s post hoc comparisons. Unpaired two-tailed Student’s *t*-test was used in the experiment in chemogenetic inhibition of the PVN-PVT neural circuit. *p* < 05 was considered statistically significant.

## Results

### The Whole-Brain BOLD Signals and Functional Connectivity Change After Activation of the Glutamatergic Neurons in Paraventricular Hypothalamic Nucleus

**FIGURE 1:**
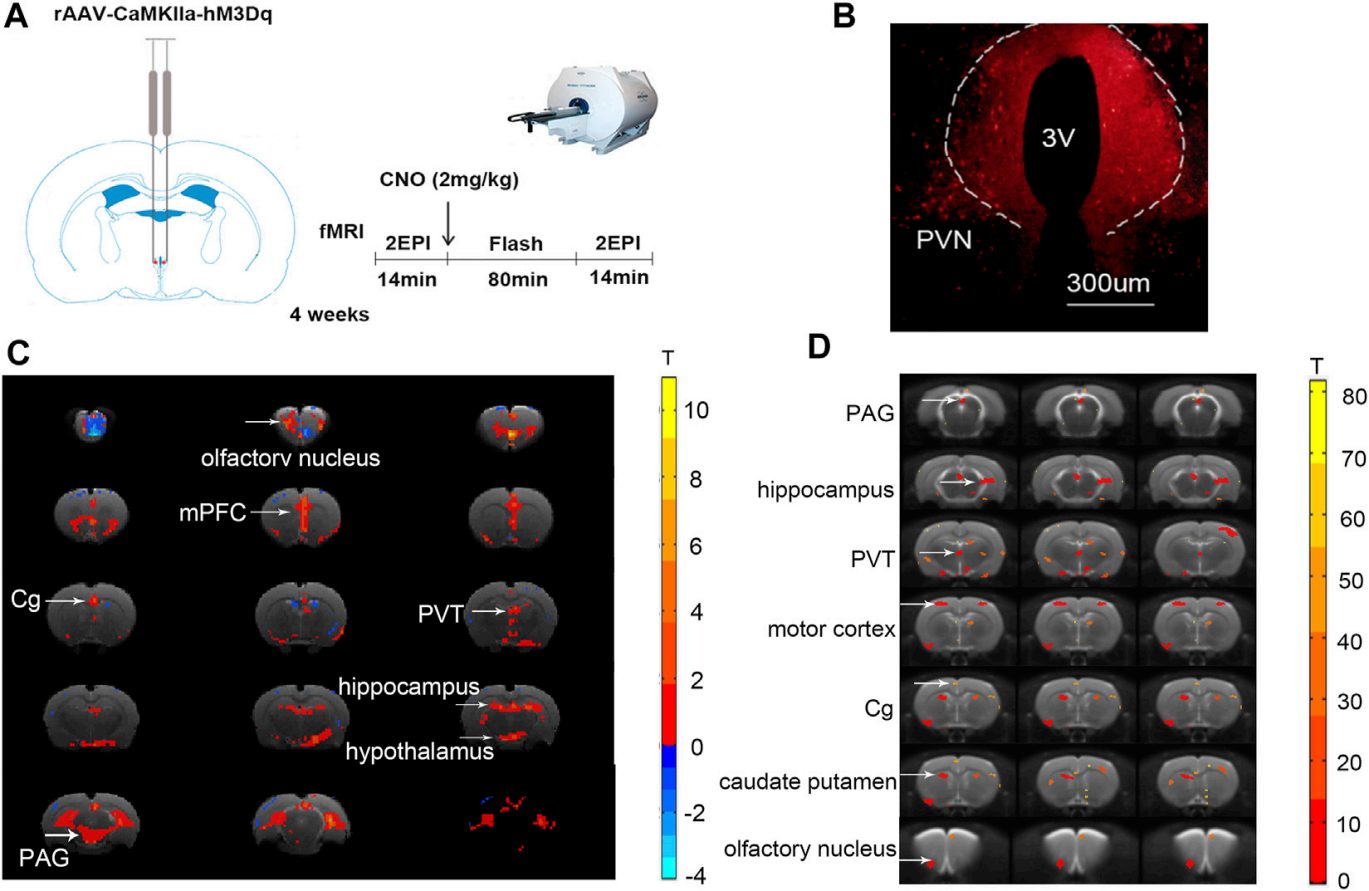
Chemogenetic activation of the PVN glutamatergic neurons alters BOLD signals and functional connectivity in the brain. **(A)** The schematic diagram of chemo-fMRI scanning. **(B)** The image showed the expression of the hM3Dq-mCherry virus in PVN. **(C)** The BOLD signals of the PVN, mPFC, Cg, PVT, PAG, and the hippocampus increased after chemogenetic activation of the glutamatergic neurons of PVN. **(D)** The functional connectivity of the PVN and the mPFC, Cg, PVT, PAG, motor cortex, caudate-putamen, hippocampus increased after chemogenetic activation of the glutamatergic neurons of PVN (*n* = 7. Paired *t*-test, FDR *p* < .05).

Using the chemo-fMRI method, after activating the PVN glutamate neurons using chemogenetics and analyzing the BOLD signal changes, we can determine functionally relevant downstream areas from the PVN. The BOLD signal changed in many brain areas after activating the glutamatergic neurons through intraperitoneal injection of CNO (2 mg kg−1). The BOLD signals of the PVN, the olfactory nucleus, the medial prefrontal cortex (mPFC), the Cg, the PVT, the periaqueductal gray nucleus (PAG), and the hippocampus increased (Figure 1C). Research has shown that the PVN neurons have reciprocal axonal projections with dozens of brain areas, including the ventromedial hypothalamus (VMH), the dorsomedial hypothalamus (DMH), the preoptic area (POA), the lateral septum (LS), the paraventricular thalamic nucleus (PVA), the lateral parabrachial nucleus (LPB), the pyramidal cell layer of the hippocampus, and the periaqueductal gray (PAG) (Luo et al., 2019). Most of these brain regions are consistent with the BOLD signals increased brain areas in our study. In addition, oxytocin (mainly produced in the PVN) in the anterior cingulate cortex attenuates neuropathic pain and emotional anxiety (Li et al., 2021), revealing that the PVN and Cg have structural or functional connections. Taking the PVN as the seed, it was found that the functional connectivity (FC) between the PVN and the olfactory nucleus, the Cg, the PVT, the PAG, the motor cortex, the caudate-putamen, and hippocampus increased after CNO injection (Figure 1D; Table 1). Among these areas, the PVT, the PAG, and the olfactory nucleus could be involved in sleep-wake control (Lu et al., 2006), suggesting that the PVN might act as a key central node for sleep-wake regulation.

**TABLE 1:** Differences between FC groups.

Brain region	Coordinate				Voxels	CL	T
	X	Y	Z				
Olfactory nucleus	33	−48		−6	68	2,297	5
Cingulate cortex	−31	40		−72	39	1,318	9
Paraventricular thalamic nucleus	−2	−32		−55	33	1,115	11
Periaqueductal gray nucleus	4	−88		−41	68	2,297	5
Motor cortex	−39	−8		−14	35	1,182	10
Caudate putamen	−25	0		−41	54	1824	6
Hippocampus	39	−64		−55	105	3,547	3

CL, cluster volume; T value, statistical value of peak. The results were corrected by FWE at the voxel level *p* < .001.

### Optogenetic Activation of the Paraventricular Hypothalamic Nucleus Glutamatergic Neurons Increases Anxiety-like Behaviors in Mice

**FIGURE 2:**
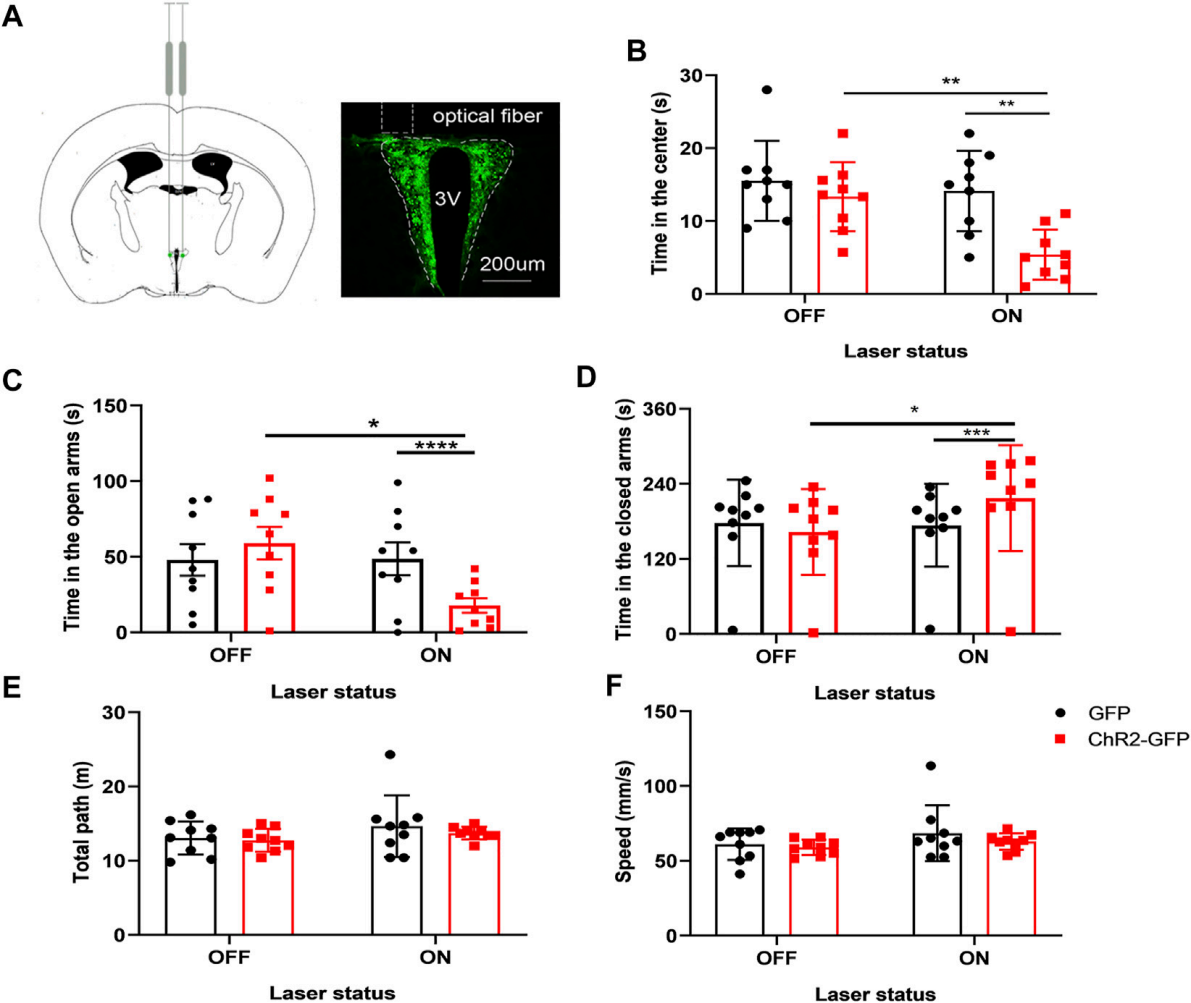
Optogenetic activation of the PVN glutamatergic neurons increases anxiety-like behaviors in mice. **(A)** The image showed the expression of the ChR2-GFP virus in PVN. **(B)** Activation of the PVN glutamatergic neurons decreased the time in the center in the open-field test. **(C)** Activation of the PVN glutamatergic neurons decreased the open-arms time in the EPM test. **(D)** Activation of the PVN glutamatergic neurons increased the close-arms time in the EPM test. **(E)** Activation of the PVN glutamatergic neurons did not affect the moving distance of the mice. **(F)** Activation of the PVN glutamatergic neurons did not affect the speed of the mice (*p < .05, **p < .01, ***p < .001, *n* = 9/group. Error bars denote SEM. Two-way repeated measures ANOVA was performed followed by Bonferroni’s *post hoc* comparisons).

To substantiate the idea that activation of glutamatergic neurons in the PVN plays a critical role in anxiety-like behaviors, rAVV-CaMKIIα-ChR2-GFP (ChR2-GFP) or rAVV-CaMKIIα-GFP (GFP) was injected into the PVN of two groups, respectively. C57 mice were randomly allocated into two groups (*n* = 9/group) according to the intra-PVN treatment (GFP or ChR2-GFP). Three weeks after bilateral virus injections, the mice underwent two well-validated unconditioned anxiety assays: the elevated plus-maze test and the open field test. We used the open-arms time, closed-arms time, and staying time in the center of the open field as the main indicators. First, without light stimulation, the two groups of mice were subjected to a baseline test for 5 min. There were no differences in the open-arms time and closed-arms time between the two groups before laser stimulation (*p* > 05; *p* > 05) (Figures 2C,D). We found that under the same conditions of light stimulation, mice in the ChR2-GFP group have less staying time in the center (Figure 2B, F1,16 = 11.30, *p* < .01), less open-arms time (Figure 2C, F1,16 = 47.56, *p* < .0001), and more closed-arms time (Figure 2D, F1,16 = 22.01, *p* < .001) than that of the GFP group. The ChR2-GFP group had less staying time in the center (Figure 2B; F1,16 = 8.977, *p* < .01), less open-arms time (Figure 2C, F1,16 = 5.078, *p* < .05), and more closed-arms time (Figure 2D, F1,16 = 5.333, *p* < .05) in the laser-on stage than that in the laser-off stage in the elevated plus-maze test. There were no statistically significant differences in moving distance and speed among each group (Figures 2E,F). The results of the experiment showed that activation of the PVN glutamatergic neurons increased anxiety-like behaviors in mice.

### Optogenetic Activation of the Paraventricular Hypothalamic Nucleus-Cingulate Cortex Neural Circuit Increases Anxiety-Like Behaviors in Mice

**FIGURE 3:**
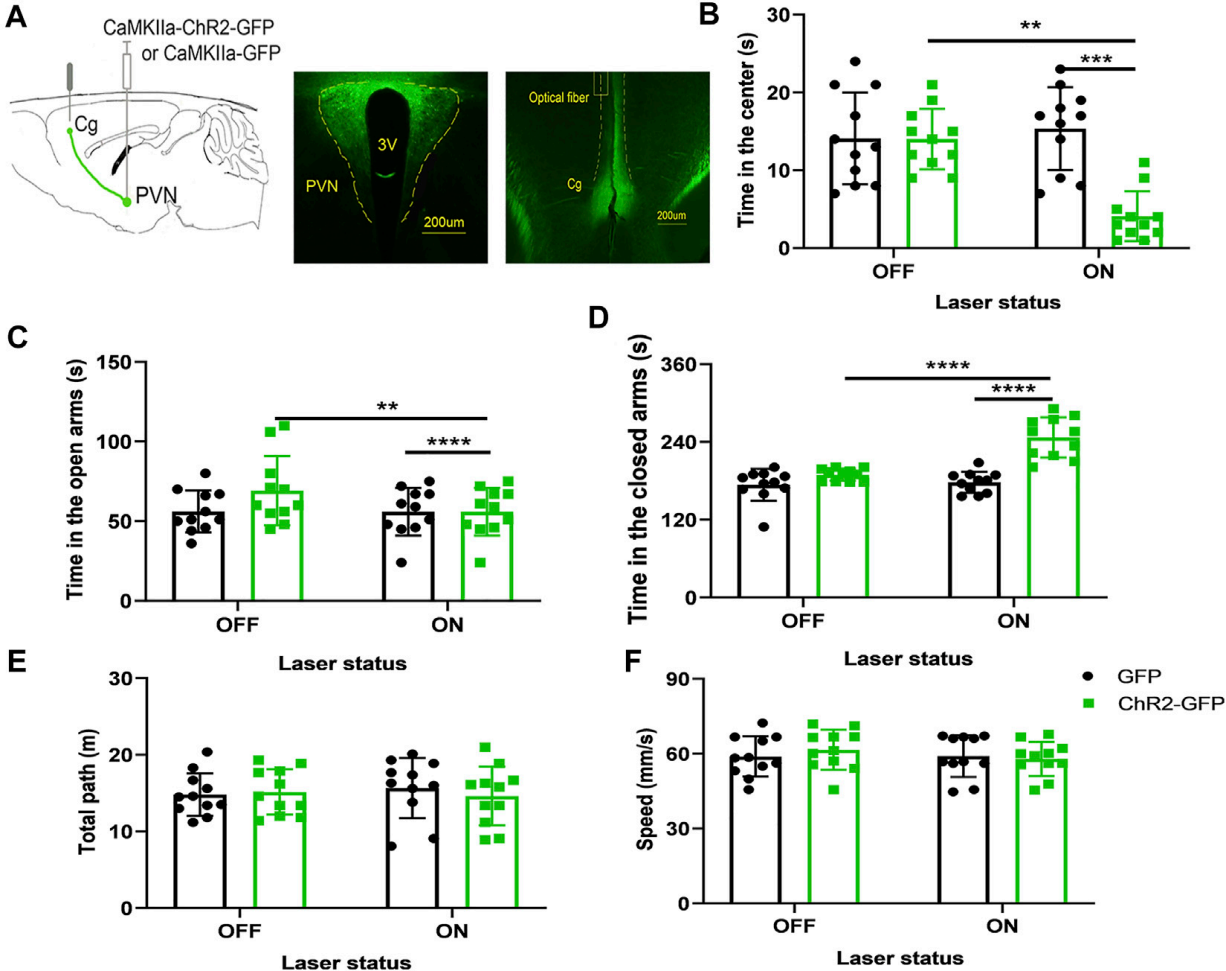
Optogenetic activation of the PVN-Cg neural circuit increases anxiety-like behaviors in mice. **(A)** The image showed the expression of the ChR2-GFP virus in PVN and ChR2-expressing glutamatergic terminals in the Cg. **(B)** Optical stimulation of PVN efferents in the Cg decreased the time in the center in the open-field test. **(C)** Optical stimulation of the PVN terminals in the Cg decreased the open-arms time in the EPM test. **(D)** Optical stimulation of the PVN efferents in the Cg increased the close-arms time in the EPM test. **(E)** Optical stimulation of the PVN efferents in the Cg did not affect the moving distance of the mice. **(F)** Optical stimulation of the PVN efferents in the Cg did not affect the speed of the mice (*p < .05, **p < .01, ***p < .001, *n* = 11/group. Error bars denote SEM. Two-way repeated measures ANOVA was performed followed by Bonferroni’s *post hoc* comparisons).

We injected ChR2-GFP and GFP optogenetic viruses into the PVN of the experimental group and control group, respectively, and embedded ferrules above the Cg brain regions, and performed behavioral experiments after 3 weeks. Mice underwent the anxiety test with the optogenetic stimulation of the PVN efferent projections to Cg. First, without laser stimulation, the two groups of mice were subjected to a baseline test for 5 min and then entered the formal test with 5 min light stimulation. We found that under the same conditions of laser stimulation, mice in the ChR2-GFP group have less open-arms time (Figure 3C, F1,20 = 123.1, *p* < .0001) and more closed-arms time (Figure 3D, F1,20 = 29.74, *p* < .0001) than that of the GFP group in the elevated plus-maze test. The ChR2-GFP group had less staying time in the center of the open field (Figure 3B, F1,20 = 15.45, *p* < .001) than that of the GFP group. In the ChR2-GFP group, the mice had less open-arms time (Figure 3C, F1,20 = 11.49, *p* < .01) and more closed-arms time (Figure 3D, F1,20 = 26.44, *p* < .0001) in the laser-on stage than that in laser-off stage. The staying time of the ChR2-GFP group in the center of the open field was also reduced by the light stimulation (Figure 3B, F1,20 = 11.44, *p* < .01). There were no statistically significant differences in moving distance and speed (Figures 3E,F). The results of this part showed that activation of the PVN efferents in the Cg increased anxiety-like behaviors in mice.

### Optogenetic Long-Term Synaptic Depression of the Paraventricular Hypothalamic Nucleus-Cingulate Cortex Neural Circuit Reduces Anxiety-Like Behaviors in Mice

In the previous study, optogenetic activation of the CeL-projecting PVT neurons increased conditioned fear expression, and CeL neurons have varied responses to optogenetic excitation of PVT terminals in the CeL: neurons with relatively high excitability (∼30%) and neurons with relatively low excitability (∼60%). While optogenetic long-term depression (LTD) induction in the CeL receiving PVT afferents effectively exerted a persistent attenuation of learned fear. The percentage of neurons with relatively high excitability was decreased by the LTD induction, and the percentage of neurons with relatively low excitability was increased by the LTD induction (Chen and Bi, 2019). These results revealed that optogenetic LTD induction of the neural pathway could induce the opposite neuronal activities and possible opposite behaviors. In this study, we also used optogenetic LTD induction to inhibit the PVN projections to the Cg. To determine whether the PVN projections to the Cg were inhibited by the LTD induction, we used 1 HZ light for 30 min to induce LTD in the Cg slices to inhibit the synapse terminals of PVN-Cg. Compared with the control slices, the LTD was successfully induced in the ChR2-LTD group. The fEPSC slope was significantly reduced in the ChR2-LTD group (*p* < .001, Supplementary Figure S1).

**FIGURE 4:**
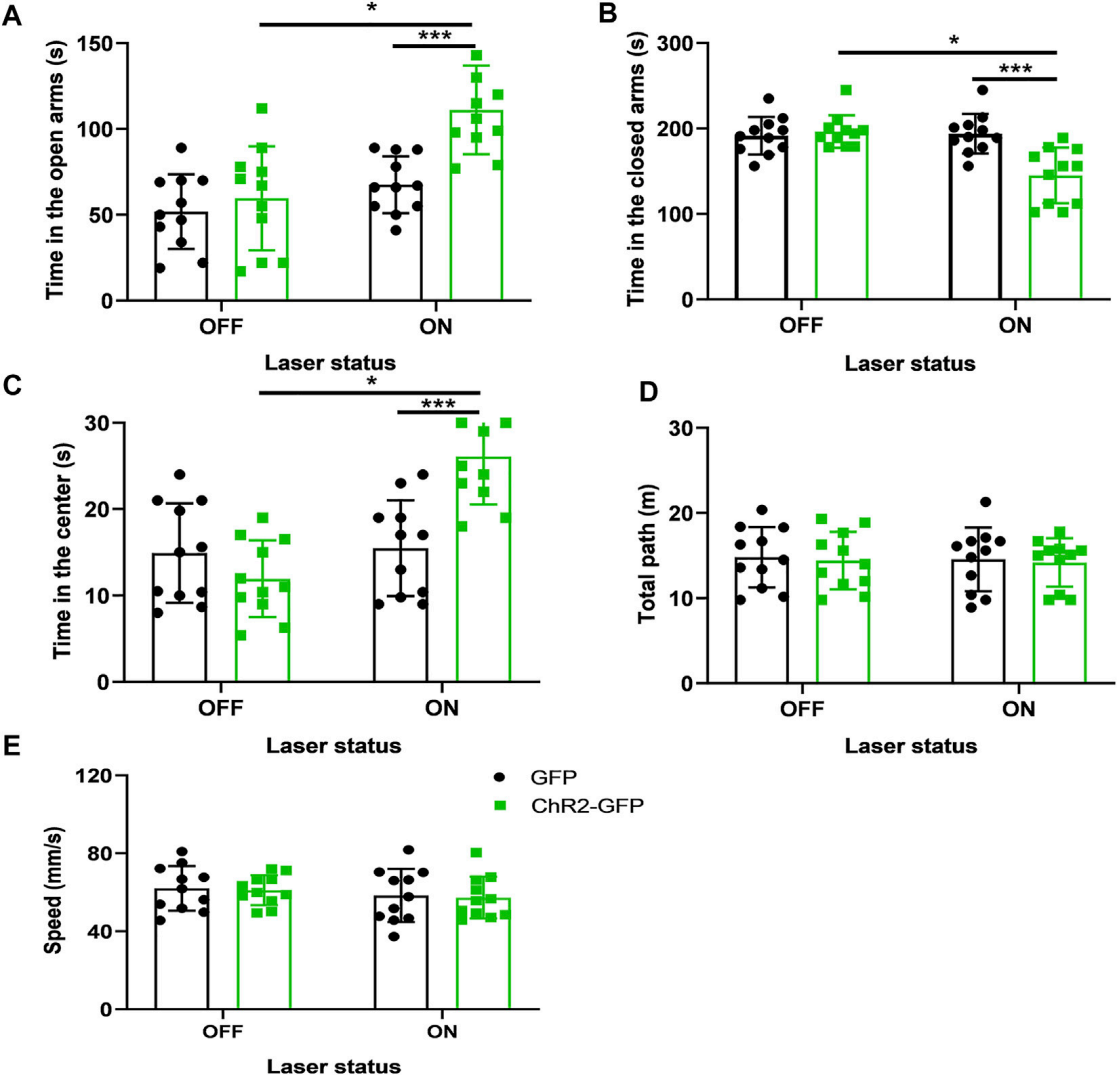
LTD of the PVN-Cg neural circuit reduces anxiety-like behaviors in mice. **(A)** Inhibition of the PVN projections to the Cg increased the open-arms time in the EPM test. **(B)** Inhibition of the PVN projections to the Cg decreased the close-arms time in the EPM test. **(C)** Inhibition of the PVN projections to the Cg increased the time in center in the open-field test. **(D)** Inhibition of the PVN projections to the Cg did not affect the moving distance of mice. **(E)** Inhibition of the PVN projections to the Cg did not affect the speed of mice (*p < .05, **p < .01, ***p < .001, *n* = 11/group. Error bars denote SEM. Two-way ANOVA test).

Next, we test the role of LTD on mice’s behavior. After 30 min of LTD, the elevated plus-maze test and open field experiment were carried out respectively. We found that under the same conditions of light stimulation, mice in the ChR2-GFP group spent more time in the open arms (Figure 4A, F_1,20_ = 20.13, *p* < .001) and less time in the closed-arms (Figure 4B, F_1,20_ = 15.02, *p* < .001) than that of the GFP group. The ChR2-GFP group had more staying time in the center of the open field than that in the GFP group (Figure 4C, F_1,20_ = 21.84, *p* < .001). In the ChR2-GFP group, the mice had more open-arms time (Figure 4A, F1,20 = 8.052, *p* < .05) and less closed-arms time (Figure 4B, F_1,20_ = 6.782, *p* < .05) in laser-on stage than that in laser-off stage in the elevated plus-maze test. The staying time of the ChR2-GFP group in the center of the open field was also increased by the light stimulation (Figure 4C, F_1,20_ = 5.382, *p* < .05). There were no statistically significant differences in the moving distance and speed of the mice between the two groups (Figures 4E,F). Taken together, these data suggested that optogenetic LTD induction of the PVN projections to the Cg reduced anxiety-like behaviors.

### Optogenetic Activation of the Paraventricular Hypothalamic Nucleus-Cingulate Cortex Neural Circuit Has No Effect on Wakefulness in Mice

**FIGURE 5:**
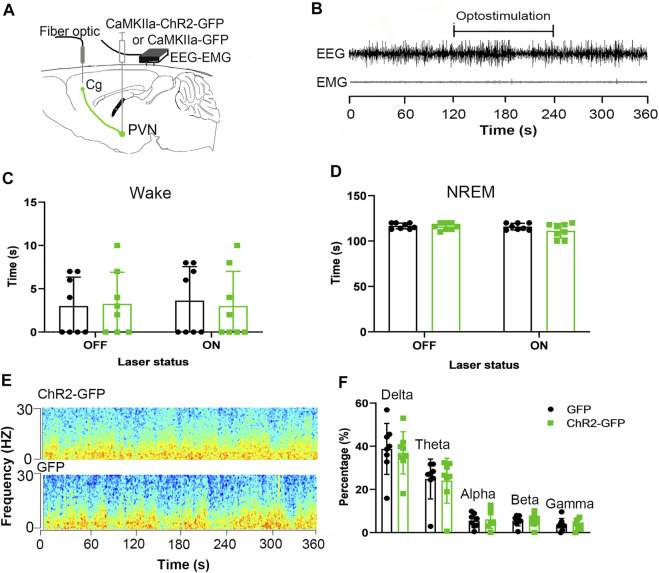
Optogenetic activation of the PVN-Cg neural circuit doesn’t affect wakefulness. **(A)** Electroencephalogram (EEG) and electromyogram (EMG) traces during optogenetic stimulation of the PVN projections to the Cg. **(B)** EEG and EMG before, during, and after the 120s light stimulation. **(C)** Activation of the PVN projections to the Cg did not affect the wake time. **(D)** Activation of the PVN projections to the Cg did not affect the NREM time. **(E)** Representative EEG power spectrum around 10 Hz stimulation delivered during NREM sleep. Color scale indicates the power (mV2) of raw power spectral density. **(F)** The percentage of delta, theta, alpha, beta, gamma of the EEG power had no significant differences (*p < .05, **p < .01, ***p < .001, *n* = 8/group. Error bars denote SEM. Two-way ANOVA test).

We injected ChR2-GFP and GFP optogenetic virus into the PVN of the experimental group and the control group, respectively, and ferrule above the Cg brain regions, and embedded sleep recording electrodes at the same time. Behavioral experiments were performed after 3 weeks. After the mice entered stable NREM sleep for 120 s, they were given optogenetic stimulation for 120 s. There were no significant differences in wake time and NREM time under the condition of 120 s light stimulation between the ChR2-GFP group and the GFP group (Figure 5B, F_1,14_ = 0.126, *p* > .05; Figure 5C, F_1,14_ = 2.127, *p* > .05). In ChR2-GFP group, there were no significant differences in wake time and NREM time between the 120s in laser-on stage and the 120s in laser-off stage (Figure 5B, F_1,14_ = 0.049, *p* > .05; Figure 5C, F_1,14_ = 2.3564, *p* > .05). This part of the experiment showed that optical stimulation of the PVN efferents to the Cg did not regulate sleep-wake behavior.

### Optogenetic Activation of the Paraventricular Hypothalamic Nucleus--Paraventricular Thalamic Nucleus Neural Circuit Increases Wakefulness

**FIGURE 6:**
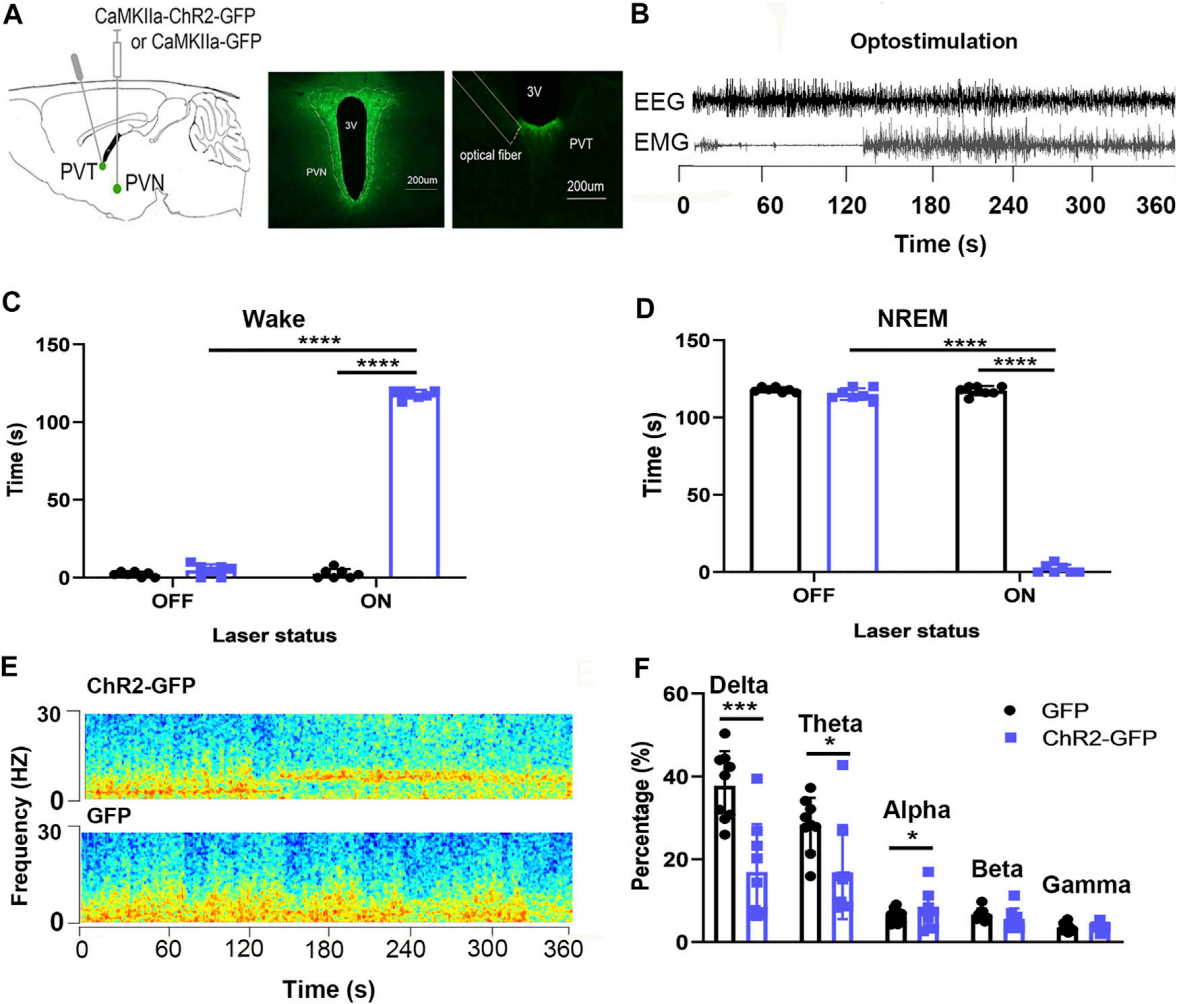
Optogenetic activation of the PVN-PVT neural circuit promotes wakefulness. **(A)** The image showed the expression of the ChR2-GFP virus in PVN and ChR2-expressing PVN glutamatergic terminals and the location of optical fiber in the PVT. **(B)** EEG and EMG traces before, during, and after the 120s optical stimulation. **(C)** Optical stimulation of PVN glutamatergic terminals to the PVT increased the wake time. **(D)** Optical stimulation of PVN glutamatergic terminals to the PVT decreased the NREM time. **(E)** Representative EEG power spectrum around 10 Hz stimulation delivered during NREM sleep. Color scale indicates the power (mV2) of raw power spectral density. **(F)** The percentage of delta in EEG power decreased after optic stimulation of PVN glutamatergic terminals to the PVT (*p < .05, **p < .01, ***p < .001, *n* = 9/group. Error bars denote SEM. Two-way repeated measures ANOVA was performed followed by Bonferroni’s post hoc comparisons).

We injected ChR2-GFP and GFP optogenetic viruses into the PVN brain regions of the experimental group and the control group mice, respectively, embedded ferrules above the PVT brain regions and embedded sleep recording electrodes at the same time. Behavioral experiments were carried out 3 weeks later. After the mice entered NREM sleep stably for 120 s, they were given PVT-specific optical stimulation for 120s. In laser-on stage, the ChR2-GFP group had more wake time (Figure 6C, F_1,16_ = 4037, *p* < .0001) and less NREM time (Figure 6D, F_1,16_ = 4459, *p* < .0001) compared with that of the GFP group. In the ChR2-GFP group, mice had more wake time (Figure 6C, F_1,16_ = 4628, *p* < .0001) and less NREM time (Figure 6D, F_1,16_ = 4385, *p* < .0001) at the 120 s period in laser-on stage compared with that 120 s in the laser-off stage. We analyzed the EEG power spectrum of ChR2-GFP group and GFP group mice under light stimulation, and found that the percentage of delta frequency in the ChR2-GFP group was significantly lower than that of the GFP group mice (Figure 6F, F_1,8_ = 31.87, *p* < .001). This part of the experiment showed that the optical stimulation of PVN projections to the PVT promoted wakefulness.

### Chemogenetic Inhibition of the Paraventricular Hypothalamic Nucleus--Paraventricular Thalamic Nucleus Neural Circuit Reduces Wakefulness

**FIGURE 7:**
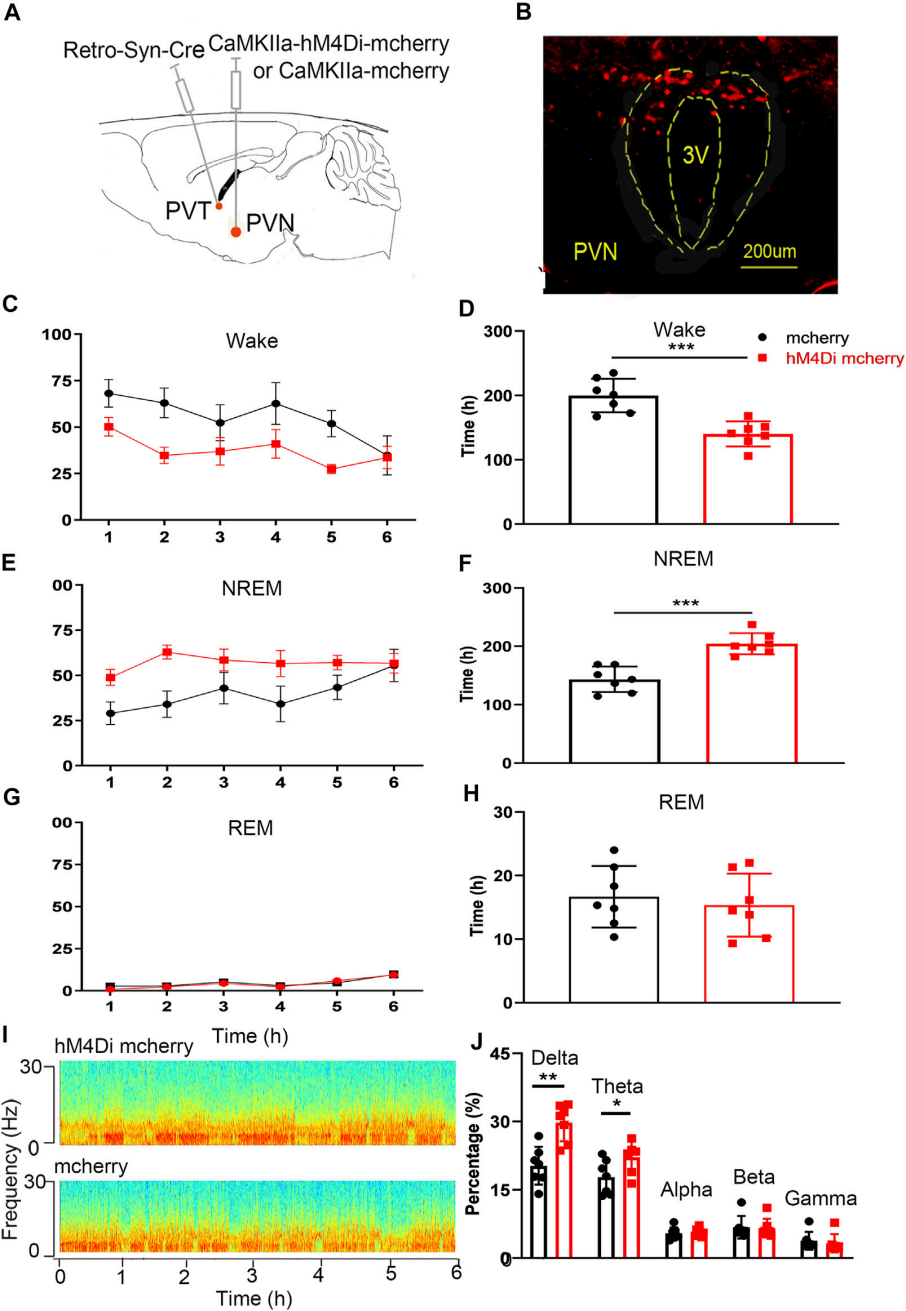
Chemogenetic inhibition of the PVN-PVT neural circuit decreases wakefulness. **(A)** Experimental design of chemogenetic inhibition of the PVT-projecting PVN neurons. **(B)** Expression of hM4Di-mCherry in PVT-projecting PVN neurons. **(C)** The wake time percentages curve during 6 h after CNO injection in hM4Di-mCherry group and mCherry group, respectively. **(D)** Chemogenetic inhibition of the PVN-PVT neural circuit decreased the wake time. **(E)** The NREM time percentages curve during 6 h after CNO injection in hM4Di-mCherry group and mCherry group, respectively. **(F)** Chemogenetic inhibition of the PVN-PVT neural circuit increased the NREM time. **(G)** The REM time percentages curve during 6 h after CNO injection in both groups. **(F)** Chemogenetic inhibition of the PVN-PVT neural circuit did not affect the REM time. **(I)** Representative EEG power spectrum in hM4Di mcherry and mcherry group. **(J)** The percentage of delta in EEG power increased after activation of the PVN-PVT neural circuit (*p < .05, **p < .01, ***p < .001, *n* = 7/group. Error bars denote SEM. Unpaired two-tailed Student’s *t*-test).

We injected rAAV-DIO-hM4Di-mcherry or rAAV-DIO-mcherry into the PVN and rAAV-retro-Syn-Cre virus into the PVT, to specifically label the PVN-PVT neural circuit, and intraperitoneal injection of CNO (0.3 mg kg−1) to inhibit the neural activities. We found that the wake time of the hM4Di-mcherry group was significantly less than that of the mcherry group (Figure 7D, *t* = 4.861, *p* < .001), and the NREM time was significantly more than that of the mcherry group (Figure 7F, *t*_12_ = 5.691, *p* < .001). There was no significant difference in the REM time between the hM4Di-mcherry group and the mcherry group (Figure 7H, *t*_12_ = .509, *p* >.05). The delta percentage of EEG in the hM4Di-mcherry group was significantly higher than that of the mcherry group (Figure 7J, *t*_12_ = 4.317, *p* < .01). This part of the experiment revealed that the chemogenetic inhibition of PVN projections to the PVT reduced wakefulness.

### Optogenetic Activation of the Paraventricular Hypothalamic Nucleus--Paraventricular Thalamic Nucleus Neural Circuit Does Not Affect Anxiety-like Behaviors in Mice

**FIGURE 8:**
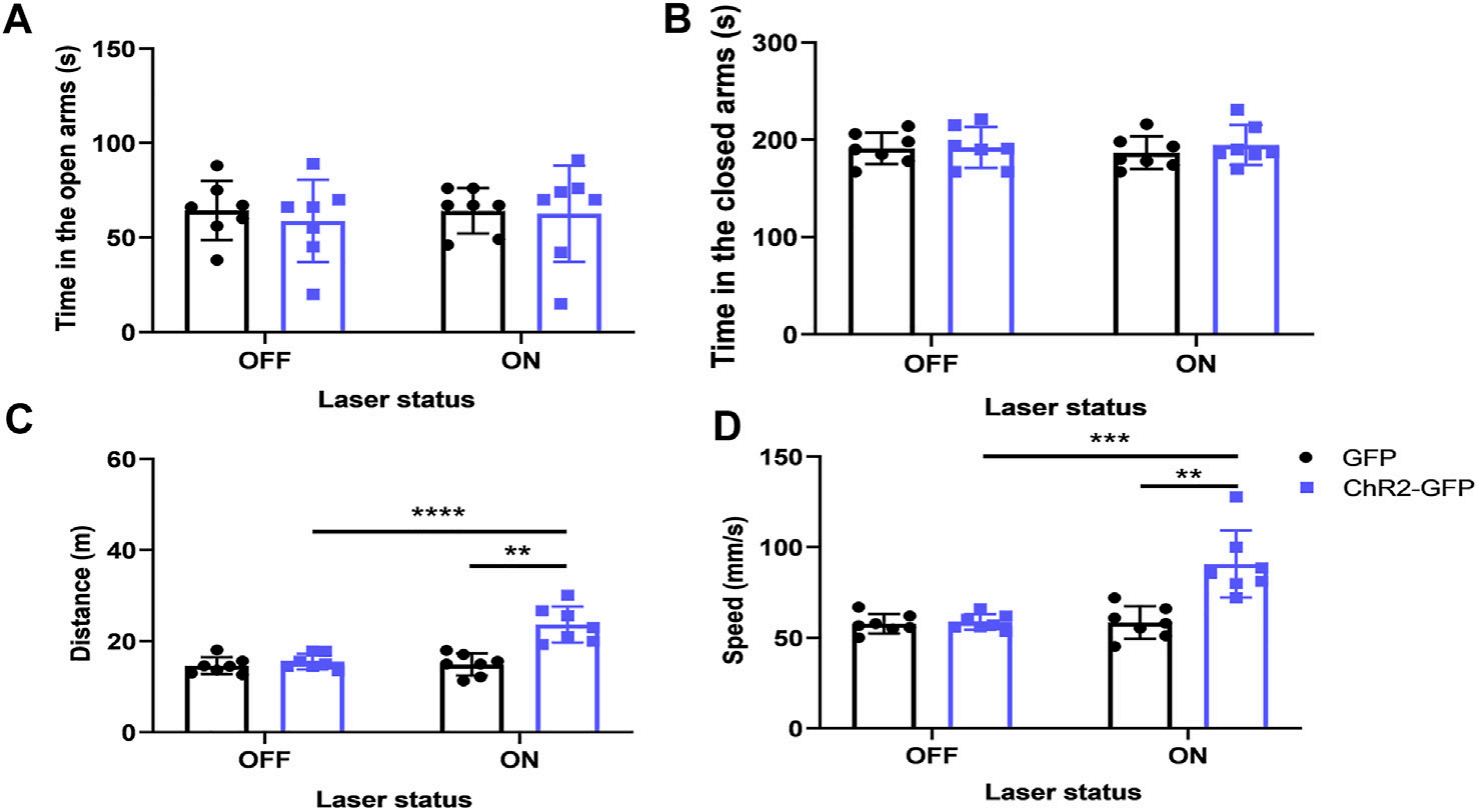
Optogenetic activation of the PVN-PVT neural circuit does not affect anxiety-like behaviors in mice. **(A)** Optical stimulation of PVN projections to the PVT did not affect the open-arms time in the EPM test. **(B)** Optical stimulation of PVN projections to the PVT did not affect the close-arms time in the EPM test. **(C)** Optical stimulation of PVN projections to the PVT increased the moving distance of the mice. **(D)** Optical stimulation of PVN projections to the PVT increased the speed of the mice (*p < .05, **p < .01, ***p < .001, *n* = 7/group. Error bars denote SEM. Two-way ANOVA test).

We injected ChR2-GFP and GFP optogenetic virus into the PVN of the experimental group and control mice respectively, and embedded ferrules in the PVT brain region for optogenetic excitation of PVN terminals in the PVT. The ChR2-GFP group showed no significant difference in the open-arms time and the closed-arms time (Figure 8A, F_1,12_ = .007, *p* > .05; Figure 8B, F_1,12_ = .001, *p* > .05) compared with that of the GFP group. Under the same conditions of laser stimulation, mice in the ChR2-GFP group had more moving distance (Figure 8C, F_1,12_ = 15.81, *p* < .01) and higher speed (Figure 8D, F_1,12_ = 12.13, *p* < .01) than that of the GFP group in the elevated plus-maze test. In the ChR2-GFP group, the mice had more moving distance (Figure 8C, F_1,12_ = 35.14, *p* < .0001) and higher speed (Figure 8D, F_1,12_ = 24.98, *p* < .001) in the laser-on stage than that in laser-off stage. These results revealed that activating the PVN-PVT pathway did not affect the anxiety-like behaviors of the mice.

## Discussion

Adequate wakefulness is fundamental for proper daytime functioning. Accumulated evidence has shown that wakefulness-promoting brain regions are involved in the neural response to stress (Cano et al., 2008). Clinical observations indicate that the paramedian region of the hypothalamus is a critical node for controlling wakefulness and stress-related behaviors. However, the specific nucleus and neural circuitry for this function remain unknown. The PVH is located in the ventral diencephalon adjacent to the third ventricle (Qin et al., 2018). Using an adeno-associated virus (AAV)-based fluorescent tracer to analyze the axon projection patterns of the PVN neurons, and find that they receive dense inputs from dozens of brain areas, including the VMH, the arcuate nucleus (Arc), the DMH, the LS, the POA, the PVA, the zona incerta (ZI), the subiculum (S), the LPB, the pyramidal cell layer of the hippocampus (py), the PAG, and the medial amygdala (MeA). Nearly all these brain areas also receive reciprocal axonal projections from PVN neurons including the POA, the DMH, the VMH, the ZI, the PVA, the PAG, the amygdala, and the LS (Luo et al., 2019). During these regions, the VMH, the LS, the POA, the PVA, the ZI, the S, the PAG, and the PB are involved in the regulation of sleep and wakefulness. The PVA, the PAG, the amygdala, the LS, the hippocampus, and the PB are involved in anxiety regulation. These results reveal that the PVN has connections with brain regions that regulate sleep-wakefulness and anxiety.

More than 90% of the PVH consists of glutamatergic neurons, whereas GABAergic neurons are more scarcely represented (Ziegler et al., 2005; Qin et al., 2018; Li et al., 2020). PVH^vglut2^ neurons co-express CRH (Zhang et al., 2020), oxytocin (OT) (Hung et al., 2017), and prodynorphin (PDYN) (Li et al., 2019). Over 90% of PVH^CRH^ neurons express vglut2 mRNA (Ziegler et al., 2005). Studies show that both the PVN^CRH^ neurons and PVN^vglut2^ neurons could regulate arousal behavior (Ono et al., 2020; C. R. Chen, et al., 2021). In addition, PVN can also regulate anxiety and depression behaviors. The HPA axis is closely related to anxiety-related behaviors, and PVN is the core brain area of the HPA axis. It is well known that the HPA axis is activated by exposure to emotional or physiological stress (Erhardt et al., 2006; Graeff, 2007; Frankiensztajn et al., 2020), and stress can increase anxiety-like behaviors in rats through activating the HPA axis activity. PVN^CRH^ neurons are considered stress neurons (Romanovet al., 2017). Optogenetic stimulation of PVN^CRH^ neurons leads to stress-induced insomnia (Li et al., 2020). Chemogenetic inhibition of the CRF neurons can attenuate sleep disorders caused by acute stress (Binder and Nemeroff, 2010; Kimura et al., 2010). Our research shows that activating the PVN glutamatergic neurons promotes wakefulness and induces anxiety-like behaviors, which is consistent with research reports. Altogether, besides the PVN^CRH^ neurons and the glutamatergic neurons being highly overlapped, we also provide evidence for their functional similarities. Our research triggered some interesting questions: such as whether the activation of glutamatergic neurons could also promote the release of CRH, whether the released CRH could deliver feed-forward excitation to the glutamatergic neurons, and so on. Further studies need to be conducted to reveal the relationship between CRH and glutamatergic neurotransmission.

Through mouse brain slices we find downstream projection targets of the PVN, including the PVT and the cingulate cortex. PVT, a midline thalamic structure that is increasingly being recognized as a critical node in the control of diverse processes such as arousal, stress, emotional memory, and motivation (Gao et al., 2020). Many studies have confirmed the obvious role of PVT in promoting arousal. Early studies have shown that the expression level of c-Fos in rats is higher in the dark environment than in the daytime (Mendoza et al., 2005; Ren et al., 2018). Using patch-clamp technology, studies have shown that the spontaneous activities of the PVT in the dark environment are stronger than that in the daytime (Kolaj et al., 2012). The optical fiber recording in freely mice shows that the calcium signal activities of PVT neurons are more active in the wake period than in the sleep period (Ren et al., 2018). In addition, optogenetic stimulation of the glutamatergic neurons in PVT promotes wakefulness from the NREM sleep, while chemogenetic inhibition of the glutamatergic neurons of PVT reduces the awake time of mice in the dark phase (Ren et al., 2018). In addition, PVT may also regulate some anxiety behaviors, for example, a recent study shows that nNOS-expressing neurons in the ventromedial prefrontal cortex can regulate anxiety behavior caused by chronic pain and excitatory neurons from PVT subregions input to the vmPFC regulate this behavior (Liang et al., 2020). In our study, optical stimulation of the PVN glutamatergic projections to the PVT promotes wakefulness but not anxiety-like behavior. Considering that PVT consists of different subregions containing functionally distinct neuronal subtypes (Gao et al., 2020). We think the subregion that the PVN glutamatergic neurons project mainly regulates arousal.

Studies have shown that the cingulate cortex is closely related to anxiety and depression (Bush et al., 2000; Vogt, 2005). ACC has also been implicated in anxiety in both human and animal studies (Frankland et al., 2004; Gross and Hen, 2004). Human imaging studies observe increased ACC activity in patients with anxiety disorders (Osuch et al., 2000), and surgical lesions or chemical inactivation in the ACC produce anxiolytic effects in humans (Hay et al., 1993)and animals (Kim et al., 2011). Other studies reveal that there exists a presynaptic form (pre-LTP) and a postsynaptic form (post-LTP) in the ACC that regulate anxiety behaviors caused by pain (Koga et al., 2015; Liang et al., 2020). In our study, optical stimulation of the PVN glutamatergic projections to the Cg regulates anxiety-like behaviors.

In conclusion, in this study, we combine chemo-fMRI, optogenetics, and chemogenetics to reveal the different neural circuit mechanisms that PVN regulates anxiety and wakefulness. From the perspective of neurological mechanisms, we will provide some insights and guidance for patients with different clinical manifestations, such as insomnia with anxiety, simple insomnia, or simple anxiety. Our research still has some aspects that need to be explored further, since the PVN is a complex nucleus that consists of different subregions containing functionally distinct neuronal subtypes, further study needs to be conducted to explore whether the PVN^vglut2^ neurons projecting to these targets belong to a common or distinct neuronal population.

## Data Availability

The original contributions presented in the study are included in the article/Supplementary Material, further inquiries can be directed to the corresponding authors.
