# Supplementary material for: Distinct Hypothalamic Paraventricular Nucleus Inputs to the Cingulate Cortex and Paraventricular Thalamic Nucleus Modulate Anxiety and Arousal
Source: Front Pharmacol. 2022 Jan 28;13:814623. doi: 10.3389/fphar.2022.814623 (PMC8832877; doi:10.3389/fphar.2022.814623)
Supplement: Supplementary file 1 [file DataSheet1.doc]

**Distinct hypothalamic paraventricular nucleus inputs to the cingulate cortex and paraventricular thalamic nucleus modulate anxiety and arousal**

Ying Liu^1+^, Bo Rao^1+^, Shuang Li^4^ , Ning Zheng^4^, Jie Wang^4*^, Linlin Bi^2, 3*^, Haibo Xu^1*^

^1^ Department of Radiology, Zhongnan Hospital of Wuhan University, Wuhan, 430071, Hubei Province, PR China.

^2^ Department of Pathology, School of Basic Medical Sciences, Wuhan University, Wuhan 430071, PR China

^3^ Wuhan University Center for Pathology and Molecular Diagnostics, Zhongnan Hospital of Wuhan University, Wuhan, 430071, PR China

^4^ State Key Laboratory of Magnetic Resonance and Atomic and Molecular Physics, Key Laboratory of Magnetic Resonance in Biological Systems, Wuhan Center for Magnetic Resonance, Wuhan Institute of Physics and Mathematics, Chinese Academy of Sciences, Wuhan, 430071, PR China

+ Ying Liu, Bo Rao contributed equally to this work and share first authorship.

*** *Corresponding author*: Jie Wang, Linlin Bi, Haibo Xu**

**E-mail addresses:** [xuhaibo1120@hotmail.com](mailto:xuhaibo1120@hotmail.com) (Hb. Xu), [linlinbi2016@whu.edu.cn](mailto:linlinbi2016@whu.edu.cn) (L. Bi), [jie.wang@wipm.ac.cn](mailto:jie.wang@wipm.ac.cn) (J. Wang).

**MATERIALS AND METHODS**

**Electrophysiological analysis**

Mice (7 weeks-old, male) were anesthetized with ketamine/xylazine (Sigma, 100/20 mg/kg, respectively, i.p.), brains were quickly removed and chilled in ice-coldmodified artificial cerebrospinal fluid (ACSF) containing (in mM): 250 glycerol, 2 KCl, 10 MgSO4, 0.2 CaCl2, 1.3 NaH2PO4, 26 NaHCO3, and 10 glucose. Mice at age 2 months or older were perfused transcardially for 2 min with modified ACSF. Coronal Cg slices (300 µm) were cut using a VT-1000S vibratome (Leica, Germany) and transferred to a chamber containing regular ACSF (in mM, 126 NaCl, 3 KCl, 1 MgSO4, 2 CaCl2, 1.25 NaH2PO4, 26 NaHCO3, and 10 glucose) at 34 °C for 30 min and at ~25°C for additional 1h before recording. All solutions were saturated with 95% O2 / 5% CO2 (vol/vol). The recording chamber was superfused (2 ml/min) with ACSF. fEPSP in Cg were recorded in current-clamp by Multi-Clamp 700B (Molecular Devices) amplifier with ACSF-filled glass pipettes (1-5 MΩ). Slices were discarded if they were unable to generate fEPSPs ≧0.8 mV or when population spikes were detected. For optogenetic LTD induction, slices were photostimulated for 30 min with trains of 473 nm light (1 Hz, 4 ms). Values were normalized to the baseline 30 min prior to LTD induction.


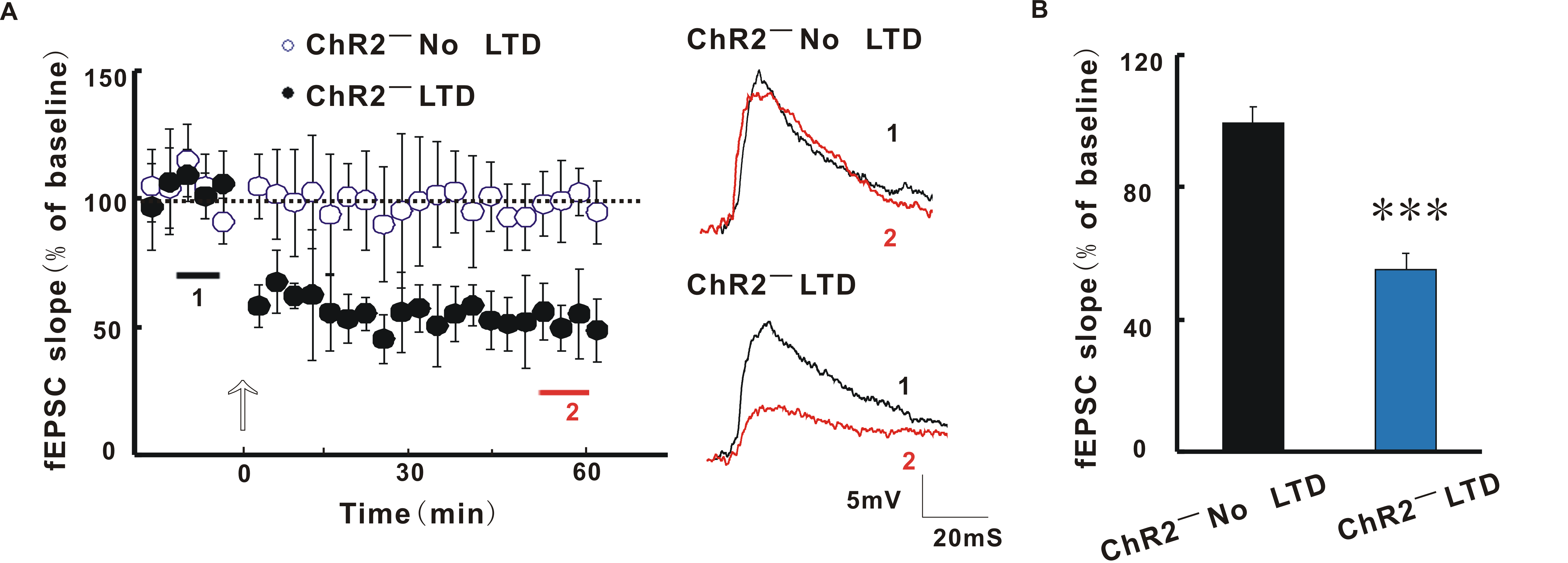


**Fig. S1** LTD induction at the Cg of AAV-ChR2-GFP mice. 3-4 weeks after the virus injection into the PVN, mice were sacrificed for the slice recording and in vitro LTD induction in the Cg. (A) Left, normalized fEPSP slopes. Dotted line, the baseline; arrow, the time of LTD induction. Right, representative traces. (B) Quantitative analysis of data (n = 6 slices, 3 mice for both groups; t-test, ***p < 0.001).
